# Association between Suicidal Ideation and Immune Parameters in First-episode Patients with Schizophrenia

**DOI:** 10.2174/011570159X384619250727094057

**Published:** 2025-08-15

**Authors:** Yu Xia, Xiaoni Guan, Meihong Xiu, Fengchun Wu

**Affiliations:** 1 Department of Emergency, The First Affiliated Hospital of Wenzhou Medical University, Wenzhou, China;; 2 Peking University HuiLongGuan Clinical Medical School, Beijing HuiLongGuan Hospital, Beijing, China;; 3 Department of Psychiatry, The Affiliated Brain Hospital, Guangzhou Medical University, Guangzhou, China;; 4 Guangdong Engineering Technology Research Center for Translational Medicine of Mental Disorders, Guangzhou, China;; 5 Key Laboratory of Neurogenetics and Channelopathies of Guangdong Province and the Ministry of Education of China, Guangzhou Medical University, Guangzhou, China

**Keywords:** Schizophrenia, suicide ideation, immune system, inflammation, NLR, immune parameters

## Abstract

**Introduction:**

Disturbed immunity and inflammation have been associated with the pathogenesis of schizophrenia (SZ) and suicidal behaviors. We hypothesized that the neutrophil-to-lymphocyte ratio (NLR) was associated with the increased risk of suicide ideation (SI) in patients with SZ.

**Methods:**

A total of 97 drug-naïve first-episode patients with SZ (15 with SI and 82 without SI) were recruited in the present study. The symptom severity was assessed using the Positive and Negative Syndrome Scale (PANSS), and differential white cell counts and SI were assessed for all patients.

**Results:**

Patients in the SI group had higher NLR values and more severe clinical symptoms than the non-SI group (all *p<*0.05). Differences remained significant when controlling for smoking, sex, BMI, and age. Logistic regression analysis showed that NLR was an independent predictor of SI in first-episode patients with SZ after controlling for clinical symptoms, smoking status, marital status, education, sex, body mass index (BMI), and age.

**Discussion:**

This study indicates a predictive role of NLR in SI at the first onset of SZ. It also suggests that monitoring NLR could be a useful clinical tool for identifying patients who may be at higher risk for SI, particularly at the onset of the disorder.

**Conclusion:**

Our findings add to the understanding of the biological factors that may contribute to SI in SZ.

## 1 INTRODUCTION

Schizophrenia (SZ) is a severe mental disorder with a high global economic burden [1]. It is reported that patients suffering from SZ have a higher mortality rate than the general population, and suicide is one of the leading causes of death in SZ [2, 3]. Suicidal behavior in individuals with SZ is an issue of public health concern. The multifaceted nature of suicide behaviors makes the management and treatment of individuals with SZ difficult.

Suicidal behavior includes suicidal ideation (SI), suicide attempts, and suicide. There is evidence that above 90% of people who have died by suicide have or had mental disorders, with SZ accounting for 2-12% of suicides [4, 5]. Recently, studies have examined potential biomarkers of suicide in patients with SZ. For example, genetic factors play a critical role in the development of suicidal behavior, with estimates of the contribution of genetic factors to suicide ranging from 45% to 69% [6]. Polymorphisms in genes such as the serotonin 2A receptor gene and brain-derived neurotrophic factor (BDNF) gene have been associated with suicidal behavior in individuals with SZ [7-9]. Studies at the molecular level have revealed a number of biomarkers related to suicidal behavior in SZ, such as the hypothalamic-pituitary-adrenal (HPA) axis and inflammatory factors [10, 11]. In addition, studies have shown that antibodies to parasites and viral infections, leptin levels, vitamin D levels, and total cholesterol (TC) may be correlated with suicidal behavior in individuals with SZ [12-15]. However, the predictive accuracy and clinical application value of these biomarkers require further investigation and validation due to confounding factors and poor consistency among studies.

The role of low-state inflammation in the pathogenesis of both suicide and SZ is a research area of increasing interest in psychiatric research. There is evidence that dysregulation of the immune system, particularly pro-inflammatory cytokines, may be involved in the pathophysiology of suicide [16, 17]. Proinflammatory cytokines have been implicated in the etiology of suicide by crossing the blood-brain barrier and activating microglia, which may contribute to neuronal damage and altered neurogenesis [18-20]. Inflammation affects neurotransmitter systems such as dopamine and glutamate [21-25], which are known to be involved in mood regulation and the pathophysiology of SZ. Autoimmune disorders, characterized by an overactive immune response, have been linked to an increased risk of SZ and suicidal behavior [16, 26]. The whole genome-wide association (GWAS) has identified genetic risk loci linked with both SZ and suicidal behavior, particularly within the major histocompatibility complex (MHC) region [27, 28]. The C4 gene, which is part of the complement pathway of the immune system, has been found to be related to an increased risk of SZ, and may also play a role in suicide risk in patients with SZ [17]. These findings suggest a complex interplay between inflammation, immune system dysregulation, and suicidal behavior in SZ.

The Immune system comprises two branches: innate and acquired immunity [29]. Notably, the neutrophil-to-lymphocyte ratio (NLR) is the ratio of cell counts from both immune pathways, including innate and acquired immunity; thereby, it is a parameter that reflects the balance between the status of both immune pathways in patients. Abnormal NLR values have been studied in mental disorders, including SZ [30-35]. Most studies reported elevated NLR values in individuals suffering from SZ compared to control subjects. Moreover, some studies have observed that individuals with suicidal behaviors, regardless of the presence of a mental disorder like SZ, tend to have higher NLR values than those without suicidal behavior [36, 37].

NLR is considered to be a reproducible and inexpensive indicator of low-grade inflammation [38-42], which can be easily calculated from the white blood cell counts under simple laboratory conditions. We hypothesized that elevated NLR at the first episode of psychosis may be a predictor of suicide ideation (SI) in SZ. Therefore, this study aimed to examine: 1) the differences in NLR values between first episode and drug naïve (FES) patients with SI and those without SI (NSI), and (2) the predictive roles of NLR values for SI in FES patients.

## 2 MATERIALS AND METHODS

### 2.1 Participants

Ninety-seven first-episode patients (55 males and 42 females) diagnosed with SZ according to the Diagnostic and Statistical Manual of Mental Disorders, 4^th^ Edition (DSM-IV-TR) criteria were enrolled in psychiatric hospitals. The diagnosis of SZ was confirmed using the SCID [43]. The inclusion criteria included: 1) 16-45 years; 2) Han Chinese ethnicity; 3) antipsychotic-naïve or cumulative exposure to antipsychotics <14 days; and 4) illness duration < 5 years. The exclusion included: 1) major medical co-morbidities; 2) abuse or drug dependence, excluding alcohol and tobacco; 3) pregnancy or breastfeeding; and 4) other major Axis I disorders. In addition, the first episode was defined in this study as the first symptom onset, as described in a previous study. The age range of 16-45 captures the majority of individuals who experienced their first episode of psychosis during this critical period.

We also recruited 65 sex- and age-matched healthy control subjects during the period of patient enrolment. Patients and controls were excluded if they were taking anti-inflammatory drugs. Subjects with ongoing infections, allergies, or a history of autoimmune disorders were also excluded.

The Institutional Review Board of the hospital approved the protocol of the study (Ethics No. 2011-4), and all patients signed an informed consent.

### 2.2 Assessment

The severity of clinical symptoms was assessed using the Positive and Negative Syndrome Scale (PANSS) [44]. Experienced psychiatrists were trained to use the PANSS scales. After training, a high inter-rater reliability was achieved (the analysis of variance (ANOVA)-ICC>0.8). The rating was administered within one week of hospital admission.

Information on patients’ history of SI was collected during the SCID review. In addition, SI data were also supplemented by family members or caregivers. A history of SI (within 2 weeks) was recorded as present (1) (the SI group), and no history of SI within the past 2 weeks as absent (0) (the NSI group).

### 2.3 Laboratory Measurements

Blood samples were collected between 7:00 and 8:00 a.m. The whole blood count was calculated using the SYSMEX XN-3000 assembly line (Sysmex Corporation, Japan). The measure was carried out in strict accordance with the operating manual. LYM and NEU count data for each patient were obtained from the hospital's laboratory electronic system.

### 2.4 Analysis

All analyses were performed in SPSS, version 20.0. The Shapiro-Wilk test and Q-Q plot were used to test normality for continuous variables. The continuous variables of normal distribution were described as mean and standard deviation (SD), and the continuous variables of non-normal distribution were described as the median and interquartile ranges (IQRs).

All patients were categorized into the SI and NSI groups. Chi-square tests, the non-parametric Mann-Whitney U test, and the Kruskal-Wallis test when comparing more than 2 groups were performed as appropriate to compare data between groups. Correlations between the demographic and clinical characteristics were investigated using Pearson’s or Spearman's correlations. The multivariate logistic regression method was performed to determine the correlation between SI and NLR values and other predictors of interest. In this model, SI was entered as the dependent variable, and NLR values, PANSS score, age, sex, BMI, smoking status, married status, and education were added as independent variables. NSI was used as a reference group. We reported correlations between variables as odds ratios (OR) and 95% confidence intervals (CI).

In addition, the area under the receiver operating characteristics (AUCROC) was applied to determine the discriminatory ability of biomarkers and associated clinical symptoms to distinguish between patients with and without SI. The ROC curve capable of distinguishing between SI and NSI was constructed based on the results of the multivariable logistic regression analyses. Sensitivity/specificity was then determined based on the ROC curves, and the optimal threshold for distinguishing between SI and NSI was subsequently determined.

## 3 RESULTS

### 3.1 Demographic and Clinical Data between the SI and NSI Groups

The median NLR values for all patients were 2.1 (IQR: 1.6, 3.3), and the median NLR values for healthy controls were 1.6 (IQR: 1.3, 2.2). NLR values were higher in FES patients compared with controls (*p=*0.003). After Bonferroni corrections, the difference remained significant.

Of all 97 patients, 15 were categorized as SI, and 82 as NSI. There was no statistical difference between SI and NSI groups in terms of age, sex, BMI, onset age, smoking status, and years of education (all *p>*0.05) (Table **1**). The rate of divorce was higher in patients with SI than those without SI (F=6.2, *p=*0.045). There were significant differences in clinical symptoms between the two subgroups. Patients in the SI group had higher general psychopathology subscores and PANSS total scores than those in the NSI group (all *p<*0.05) (Table **1**).

### 3.2 Comparisons of White Cell Counts between the Two Subgroups

Patients in the SI group had higher NLR and NEU counts than the NSI group (*p=*0.002 and *p=*0.003). After controlling for age, BMI, smoking status, and sex, the differences were also present (all *p<*0.05). However, there was no significant difference in LYM counts between the SI and NSI groups (*p>*0.05).

In the SI group, BMI was associated with NLR (r=-0.55, *p=*0.04) and LYM counts (r=0.64, *p=*0.01) (Table **2**). There was no significant association between age, onset age, education years, smoking status, and white cell counts in the SI group (all *p>*0.05). In contrast, in the NSI group, smoking patients had higher NLR values than non-smoking patients (F=4.3, *p=*0.04). No significant associations were observed between white blood counts and BMI or age, onset age, and education years (all *p>*0.05).

### 3.3 Associations between SI and White Cell Counts and Symptom Severity in Patients

In the SI group, LYM counts were negatively associated with PANSS-N subscore (r=-0.61, *p=*0.02). In the NSI group, there was a significant correlation between NLR and PANSS-G subscore (r=0.28, *p=*0.01) and the total score (r=0.26, *p=*0.02), as well as a significant association between NEU counts and PANSS N subscore (r=0.29, *p=*0.008), G subscore (r=0.25, *p=*0.03) and total scores (r=0.37, *p=*0.001).

Logistic regression analysis using IS as a dependent variable was performed to identify risk factors correlated with SI in SZ. We found that after controlling for clinical symptoms, age, sex, BMI, education, marriage status, and smoking status, NLR was a predictive biomarker for SI in patients with SZ (β=0.476, *p=*0.046, OR=1.610, 95%CI: 1.008 to 2.572) (Table **3**). The logistic regression model was statistically significant (*X*^2^= 35.77; *p<*0.001).

In addition, AUCROC was constructed to identify SI and NSI and showed the AUC value of NLR was 0.720 (95% CI: 0.574 to 0.865) to distinguish SI from NSI (*p=*0.007). According to the ROC curve, the best cut-off value for the NLR to distinguish SI from NSI was 2.66 (sensitivity: 66.7%, specificity: 59.8%).

## 4 DISCUSSION

To the best of our knowledge, this study is the first to investigate the role of NLR in predicting SI in FES patients with SZ. We found that NLR values were higher in the SI group than in the NSI group. In addition, after controlling for many confounders, including age, sex, smoking status, marital status, education, and BMI, we found that NLR values predicted SI in FES patients with SZ.

In this study, SZ patients with SI had higher NLR values than those without SI. This finding is in line with previous studies in other neuropsychiatric disorders [45-47], suggesting that immune abnormalities and inflammation may play a role in the pathogenesis of SI in patients with SZ. Notably, the relationship between inflammation and NLR values is multifaceted and may be influenced by various variables. There is evidence that smoking, age, sex, family relationships, substance abuse, obesity, chronic disease, acute stress response, and antipsychotic treatment contribute to transient increases in NLR values. Our study recruited FES patients, which minimizes the potential influence of age, chronic disease, and antipsychotic medication. In addition, patients with and without SI were well matched on some confounding factors, including smoking, education, sex, and obesity. It is noted that the SI group had more severe symptoms than the NSI group, as well as different marital statuses. The results remained significant after controlling for these factors. The difference in NLR values may highlight the potential use of NLR as a biological predictor or biomarker for identifying SZ patients who are at greater risk of suicide.

We also found that patients in the SI group had more severe general psychopathology as measured by the PANSS than those in the NSI group. In line with our findings, previous studies have shown that SZ patients with suicidal behavior may experience more intense positive symptoms, such as hallucinations, delusions, and thought disorders [48-54]. Other studies have also observed higher general psychopathology scores in patients with SI than those without SI [55-57]. The general psychopathology measured by the PANSS scale mainly consists of depression, anxiety, stress, hopelessness, insomnia, and guilt, which are common in patients with SI [55, 58, 59]. Patients with suicidal tendencies may score higher in this subscale due to the presence of these symptoms. We speculate that the potential mechanism may be due to the fact that individuals with SZ often experience pronounced emotional and psychological distress, as well as somatic concerns such as physical discomfort or pain, contributing to a higher score in the PANSS-G subscore.

Despite significant advancements in mental health care, suicide remains a major contributor to death among patients with SZ. The identification and application of biological markers are thought to hold promise for improving prevention and treatment strategies for suicidal behaviors. Interestingly, we found that patients with SZ who also experience SI tend to have higher NLR values, and NLR may be indicative of a risk for suicidal behavior in this study. Although the exact mechanism is not fully understood, inflammation is believed to be involved in the pathophysiology of SZ and the associated suicidal behavior. This study aligns with previous studies that have shown a correlation between increased systemic inflammation and a higher prevalence of SI among individuals with SZ [16, 60, 61]. Consistent with our findings, studies have shown that NLR was a potentially measurable biomarker of suicide in patients with major depression [46, 62]. Stress and depression have been shown to increase NEU counts, but decrease LYM counts [63], leading to increased NRL values. Indeed, studies have suggested that NLR is a reliable marker of inflammation and that the NLR values in patients with SZ or individuals with suicidal behaviors are higher compared to the general population, suggesting a state of chronic inflammation. Our study is the first to establish a potential link between SI and NLR values, a significant step in understanding the complex interplay of inflammation and suicide risk. If our finding was verified by other studies, it could guide clinical practice by helping to identify individuals who may benefit from closer monitoring and targeted interventions. Notably, while NLR holds immense promise as a biomarker for suicide ideation in SZ, it is not yet a definitive or standalone indicator. NLR is just one of many potential biomarkers, and further research is needed to establish its predictive validity and to understand the underlying mechanisms linking inflammation with suicidal behavior in individuals with SZ. On the other hand, other biological markers, such as genetic markers, biochemical markers, and neuroimaging biomarkers, have also been found to be in relation to suicidal behaviors in patients with SZ. Further research is warranted to clarify the role of NLR and other biological risk factors in predicting SI in SZ, which may provide a deep understanding of the complex interrelationship between biological risk factors and SI in SZ.

This study had several main limitations. First, studying the first episode of SZ without or with minimal antipsychotic exposure is particularly challenging, especially studying patients with suicidal behaviour. This is because the rate of suicide behaviors is low, just 2-12%. Therefore, the sample size of patients with SI was small (n=15) in this study, which may introduce bias. In addition, small samples can lead to high variability in the results and may not be representative of the larger population, affecting the generalizability of the findings. Our study was a pilot study and it provided preliminary research findings. Second, not all potential risk factors that can alter the inflammatory response have been taken into account in this study. These factors include social factors (*e.g*., interpersonal stressors including non-heterosexual orientation, bullying, and persecution), negative life events (*e.g*., abuse, family violence), traumatic life events in adulthood, psychological resilience, unemployment, and economic stress. Third, some patients may not express SI in the interview due to fear of stigma, lack of trust in the interviewer, and fear of involuntary hospitalization. To address this issue, the assessment of SI in SZ should be based on a comprehensive evaluation that includes clinical symptoms, personal and family history of suicidal behavior, and the presence of comorbidities such as depression. Fourth, all participants were Chinese. Our results were unable to be generalized to other ethnic or geographical populations. Fifth, NLR is a multifactorial biomarker that can be influenced by various clinical and behavioral factors, including acute impulsive aggressive behavior. Future research should consider incorporating measures of impulsive aggressive behavior to better control for this potential confounding factor. This would allow for a more accurate interpretation of the relationship between NLR and suicidal ideation. Sixth, the potential use of NLR as a prognostic marker for SI needed to be carefully interpreted, especially in patients with co-existing cardiovascular diseases, infections, or other inflammatory conditions. Future research should address these limitations by exploring the specificity of NLR for SI and considering the broader clinical context in which it is measured. Seventh, NLR values for the NSI group were not dramatically different from those reported for the general population and healthy controls [64], which underscores the importance of careful interpretation of NLR as a biomarker.

## CONCLUSION

This is the first study to show that the NLR may be a predictor of SI in patients with SZ after controlling for many confounding factors. The NLR is a simple and low-cost measure that is usually performed as part of routine testing in psychiatric hospitals. Measuring NLR may help identify individuals at higher risk for SI, leading to more targeted interventions and treatment plans for patients with SZ. If our findings can be replicated, NLR could be developed as a useful biomarker for SI in clinical practice, particularly in first-episode patients with SZ. It is important to note that NLR is only one among several biomarkers being investigated for their potential predictive value in suicide risk. Further research is warranted to validate the relationship between SI and NLR values in FES patients using a longitudinal design and to determine its clinical utility.

## Figures and Tables

**Table 1:** Demographic characteristics and clinical data in first-episode patients with or without suicide ideation.

**Variable**	**NSI (n=82)**	**SI (n=15)**	**F, Z or *X*^2^ (p)**
Gender (M/F)	47/35	8/7	0.1(0.78)
Age (ys)	24.2 ± 7.0	23.5 ± 6.4	0.1(0.72)
Education (ys)	8.2 ± 2.7	7.7 ± 3.1	0.3(0.58)
BMI (kg/m^2^)	20.4 ± 2.9	19.6 ± 2.3	1.2(0.28)
Smoking status	-	-	2.2(0.19)
Yes (n, %)	18 (22.0)	6 (40.0)	-
No (n, %)	64 (78.0)	9 (60.0)	-
Marriage status	-	-	6.2(0.045)
Single (n, %)	62(75.6)	10(66.7)	-
Married (n, %)	19(23.2)	3(20)	-
Divorced (n, %)	1(1.2)	2(13.3)	-
Age of onset (ys)	23.5 ± 7.0	22.7 ± 6.8	0.2(0.67)
***Clinical Symptoms (PANSS)***			
Positive subscale	18.9 ± 4.9	21.0 ± 4.5	2.4(0.12)
Negative subscale	18.1 ± 7.0	20.6 ± 7.2	1.7(0.20)
General subscale	31.8 ± 6.9	37.5 ± 6.9	8.8(0.004)
Total score	68.5 ± 13.1	78.5 ± 12.9	7.5(0.007)
***White Cell Counts***			
NEU (×10^9^)	4.3 ± 1.6	5.6 ± 1.6	9.5(0.003)
LYM (×10^9^)	2.1 ± 0.8	1.9 ± 0.9	0.7(0.40)
NLR	2.1(1.5, 3.1)	3.3(1.9, 5.1)	2.7(0.007)

**Abbreviation:** *M* male; *F* female; *ys* years; *BMI* Body mass index; *PANSS* the positive and negative syndrome scale.

**Table 2:** Associations between demographic data, clinical symptoms, white cell counts, and SI.

-	NLR				NEU				LYM			
	SI		NSI		SI		NSI		SI		NSI	
	r	p	r	p	r	p	r	p	r	p	r	p
Age(y)	-0.27	0.34	0.002	0.99	-0.23	0.41	-0.08	0.48	0.10	0.72	-0.22	0.05
Onset age(y)	-0.15	0.60	0.02	0.89	-0.24	0.40	-0.06	0.62	-0.01	0.96	-0.21	0.06
Education(y)	-0.38	0.19	0.08	0.47	-0.23	0.43	0.03	0.81	0.34	0.23	-0.02	0.87
BMI(Kg/m^2^)	-0.55	0.04	-0.06	0.61	0.01	0.97	0.03	0.78	0.64	0.01	0.04	0.89
P subscore	0.07	0.80	0.12	0.28	-0.23	0.40	0.19	0.08	-0.26	0.34	0.11	0.29
N subscore	0.46	0.09	0.18	0.11	-0.05	0.86	0.29	0.008	-0.61	0.02	0.06	0.59
G subscore	0.18	0.53	0.28	0.01	-0.16	0.58	0.25	0.03	-0.36	0.19	-0.10	0.39
total score	0.22	0.43	0.26	0.02	-0.15	0.60	0.37	0.001	-0.39	0.16	0.06	0.57

**Abbreviations**: SI suicide ideation; BMI Body mass index; y years; LYM lymphocytes; NEU neutrophils; NLR neutrophil/lymphocyte ratio; PANSS Positive and Negative Syndrome Scale, P positive symptom, N negative symptom, G the general psychopathology subscale.

**Table 3:** Regression analysis of the associations between suicide ideation (SI), NLR, and clinical symptoms.

**Logistic Regression Analysis**						
**With SI or Not**		**-**				
**-**	**B**	**SE**	**Wals**	**Sig.**	**Exp (B)**	**Exp (B) 95% CI**
Sex (male/female)	0.911	0.775	1.381	0.240	2.486	0.544-11.354
Age (y)	-0.129	0.089	2.125	0.145	0.879	0.739-1.045
Marriage status	1.317	0.890	2.191	0.139	3.732	0.652-21.351
Education	0.007	0.131	0.003	0.957	1.007	0.78-1.301
Smoking status	0.777	0.793	0.961	0.327	2.176	0.460-10.298
BMI (kg/m^2^)	0.014	0.134	0.011	0.918	1.014	0.780-1.318
PANSS score	0.033	0.026	1.622	0.203	1.033	0.983-1.087
NLR values	0.476	0.239	3.975	0.046	1.610	1.008-2.572

**Abbreviation:** SI suicide ideation; BMI Body mass index; y years; NLR neutrophil/lymphocyte ratio; PANSS Positive and Negative Syndrome Scale.

## Data Availability

All data generated or analyzed during this study are included in this published article.

## LIST OF ABBREVIATIONS

ANOVA: The Analysis of Variance
AUCROC: The Area Under the Receiver Operating Characteristics
BDNF: Brain-derived Neurotrophic Factor
BMI: Body Mass Index
CI: Confidence Intervals
DSM-IV-TR: Diagnostic and Statistical Manual of Mental Disorders, 4th Edition
GWAS: Whole Genome-wide Association
HPA: Hypothalamic-pituitary-adrenal
IQRs: Interquartile Ranges
MHC: Major Histocompatibility Complex
NLR: Neutrophil-to-lymphocyte Ratio
PANSS: The Positive and Negative Syndrome Scale
SI: Suicide Ideation
SZ: Schizophrenia
TC: Total Cholesterol

## STANDARDS OF REPORTING

STROBE guidelines were followed.
